# Supplementary material for: Pangenomics of the Symbiotic Rhizobiales. Core and Accessory Functions Across a Group Endowed with High Levels of Genomic Plasticity
Source: Microorganisms. 2021 Feb 16;9(2):407. doi: 10.3390/microorganisms9020407 (PMC7920277; doi:10.3390/microorganisms9020407)
Supplement: Supplementary file 1 [file microorganisms-09-00407-s001.zip › Supplementary_Figures S1-S3.docx]

**Supplementary Figures**

Pangenomics of the symbiotic Rhizobiales. Core and accessory functions across a group endowed with high levels of genomic plasticity.

**Riccardo Rosselli, Nicola Laporta , Rosella Muresu, Piergiorgio Stevanato, Giuseppe Concheri and Andrea Squartini**


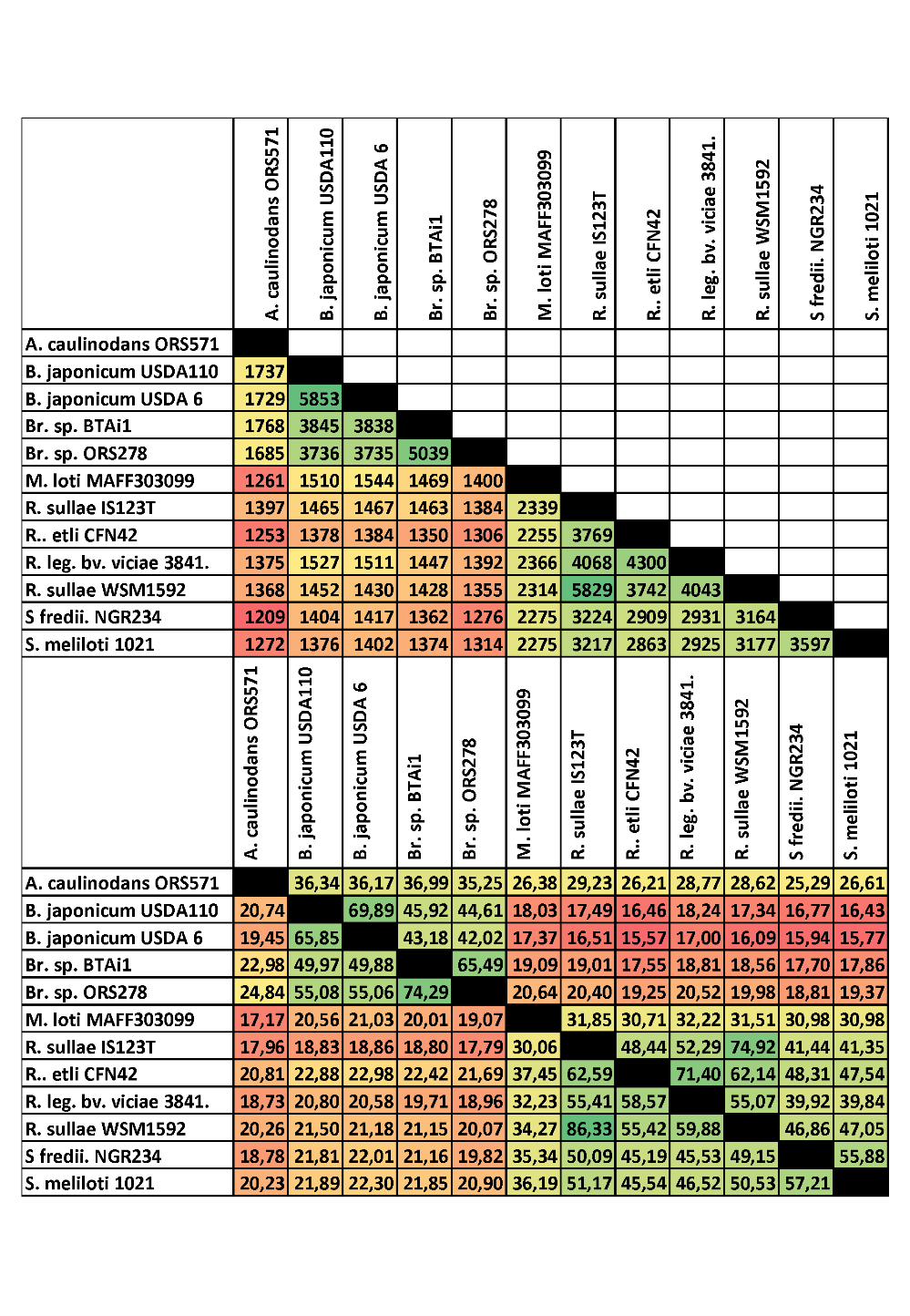


**Fig. S1. Pairwise (two-taxa) core genomes.** Top table: number of shared genes between each pair. Bottom table: Percent of own's genome; In the horizontal dimension: percent of the genome of each taxon listed in rows which shares genes with taxa listed in columns. In the vertical dimension: percent of the genome of each taxon listed in columns which shares genes with taxa listed in rows. (E.g. the core set of 1737 in the *A.caulinodans* ORS571- *B. japonicum* USDA110 pair, represents 36.34 % of the *A. caulinodans* ORS571 genome and 20.74 % of the *B. japonicum* USDA110 genome).


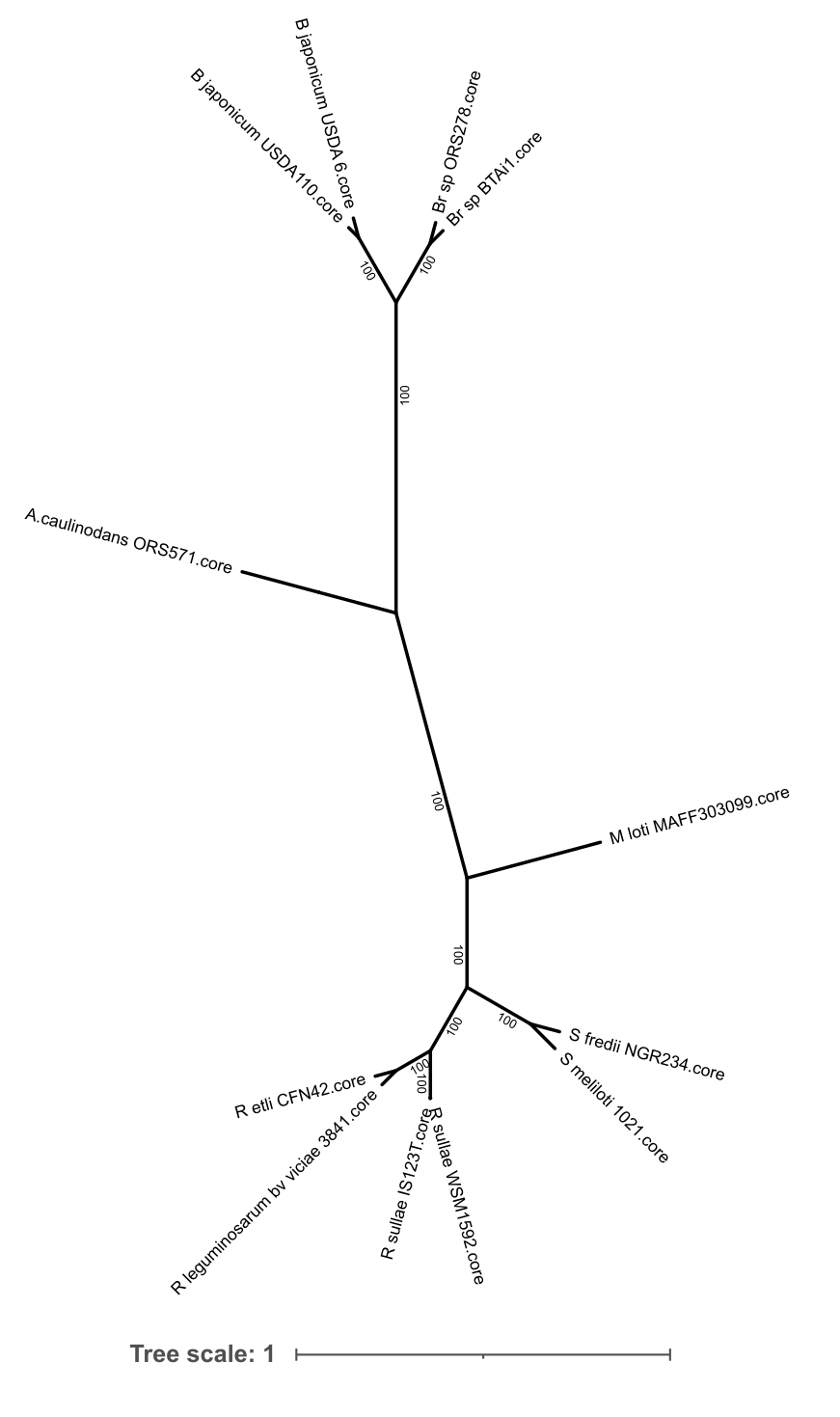


**Fig. S2** Unrooted phylogenomic topology showing relationships between Rhizobiales basing on core-gene comparisons.


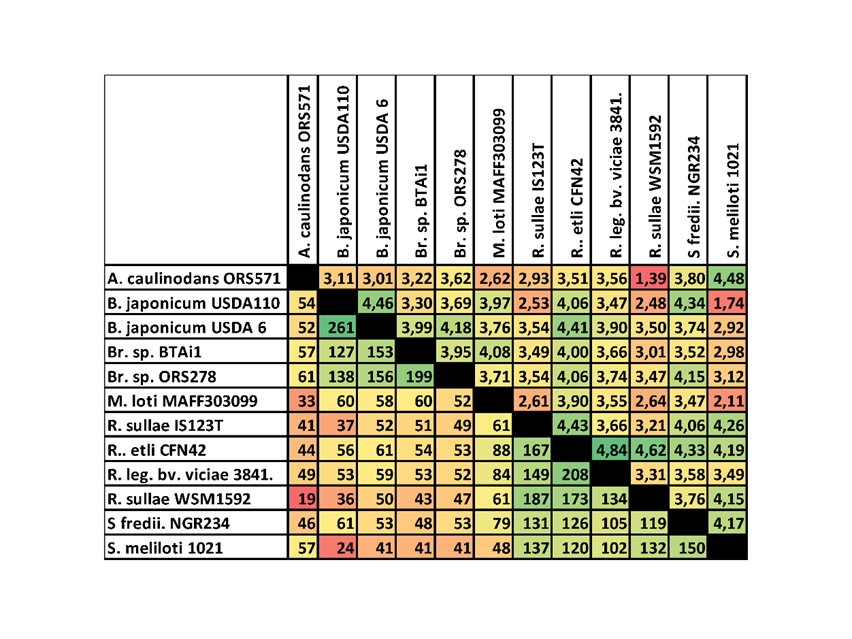


**Fig. S3. Functionally diverged genes.** The number of cases observed in each pairwise comparison is reported on the bottom–left triangle. The corresponding percentage of the total number of shared genes (shown in Fig.S1) is reported on the top-right triangle.
